# Using osmotic stress to stabilize mannitol production in *Synechocystis* sp. PCC6803

**DOI:** 10.1186/s13068-020-01755-3

**Published:** 2020-07-02

**Authors:** Wenyang Wu, Wei Du, Ruth Perez Gallego, Klaas J. Hellingwerf, Aniek D. van der Woude, Filipe Branco dos Santos

**Affiliations:** 1 Molecular Microbial Physiology Group, Swammerdam Institute for Life Sciences, Faculty of Science, University of Amsterdam, Science Park 904, 1098 XH Amsterdam, The Netherlands; 2 Photanol B.V, Matrix V, Science Park 406, 1098 XH Amsterdam, The Netherlands; 3 Present Address: NIOZ Royal Netherlands Institute for Sea Research, Department of Marine Microbiology and Biogeochemistry, Utrecht University, P.O. Box 59, Den Burg, Texel, 1790 AB Utrecht, The Netherlands

**Keywords:** (D-)Mannitol, *Synechocystis* sp. PCC6803, Compatible solutes, Production stability, Salt stress

## Abstract

**Background:**

Mannitol is a C(6) polyol that is used in the food and medical sector as a sweetener and antioxidant, respectively. The sustainable production of mannitol, especially via the direct conversion of CO_2_ by photosynthetic cyanobacteria, has become increasingly appealing. However, previous work aiming to achieve mannitol production in the marine *Synechococcus* sp. PCC7002 via heterologous expression of mannitol-1-phosphate-5-dehydrogenase (*mtlD*) and mannitol-1-phosphatase (*m1p*, in short: a ‘mannitol cassette’), proved to be genetically unstable. In this study, we aim to overcome this genetic instability by conceiving a strategy to stabilize mannitol production using *Synechocystis* sp. PCC6803 as a model cyanobacterium.

**Results:**

Here, we explore the stabilizing effect that mannitol production may have on cells faced with osmotic stress, in the freshwater cyanobacterium *Synechocystis* sp. PCC6803. We first validated that mannitol can function as a compatible solute in *Synechocystis* sp. PCC6803, and in derivative strains in which the ability to produce one or both of the native compatible solutes was impaired. Wild-type *Synechocystis*, complemented with a mannitol cassette, indeed showed increased salt tolerance, which was even more evident in *Synechocystis* strains in which the ability to synthesize the endogenous compatible solutes was impaired. Next we tested the genetic stability of all these strains with respect to their mannitol productivity, with and without salt stress, during prolonged turbidostat cultivations. The obtained results show that mannitol production under salt stress conditions in the *Synechocystis* strain that cannot synthesize its endogenous compatible solutes is remarkably stable, while the control strain completely loses this ability in only 6 days. DNA sequencing results of the control groups that lost the ability to synthesize mannitol revealed that multiple types of mutation occurred in the *mtlD* gene that can explain the disruption of mannitol production.

**Conclusions:**

Mannitol production in freshwater *Synechocsytis* sp. PCC6803 confers it with increased salt tolerance. Under this strategy, genetically instability which was the major challenge for mannitol production in cyanobacteria is tackled. This paper marks the first report of utilization of the response to salt stress as a factor that can increase the stability of mannitol production in a cyanobacterial cell factory.

## Background

Mannitol is a six-carbon sugar alcohol with multiple biological applications, such as a sweetener and an antioxidant. Therefore, mannitol has been widely applied in the food-, pharmaceutical-, and chemical industry, and it is of high commercial value [1]. Recently, cyanobacteria have gained much attention to be developed as photosynthetic cell factories to convert CO_2_ directly into biochemical compounds of interest. Enabled by genetic engineering, the production of many different compounds [2–8] (e.g., ethanol, lactate, terpenes to name only a few) has already been achieved in different cyanobacterial species. Given the commercial value of mannitol and our current need for green alternatives, sustainable production of this sweetener by cyanobacteria is therefore becoming increasingly attractive.

The first report of mannitol production directly from CO_2_ in the marine cyanobacterium *Synechococcus.*sp PCC7002 (hereafter, *Synechococcus*) appeared in 2014 [9]. This production was achieved via heterologous expression of the genes encoding mannitol-1-phosphate-5-dehydrogenase (*mtlD*) and mannitol-1-phosphatase (*m1p*) either as individual proteins [9] or as a fused protein [10], to convert (part of) the endogenous metabolite fructose-6-phosphate into mannitol. This two-step conversion from fructose-6-phosphate to mannitol is in principle superior to the single-step conversion of fructose to mannitol via mannitol dehydrogenase (*mdh*). This is due to the fact that in cyanobacteria, fructose-6-phosphate is more abundant comparing to fructose, since fructose-6-phosphate is one of the main metabolites in the Pentose Phosphate Pathway that carries a high metabolic flux under photoautotrophic conditions. Hence, more mannitol would be expected to be produced via this two-step conversion, though the accumulation of the intermediate mannitol-1-phosphate might be harmful for the cells resulting in genetic instability problems [11]. In the study of Jacobsen and Frigaard, a concentration of mannitol of 1.1 g L^−1^ was reached after 12 days, with an average productivity of 0.15 g L^−1^ day^−1^ [9]. However, this production system turned out to be genetically unstable, possibly because the heterologous mannitol production pathway directly competes for metabolic intermediates with biomass formation [12], which imposes a fitness burden on the mannitol producing cells. These strains thus become susceptible to suppressor mutations, such as insertions or deletions, that would lower/remove this fitness burden. In the study of Jacobsen and Frigaard [9], the genetically engineered *Synechococcus* strains suffered from both incomplete genome segregation and from suppressor mutations occurring in the *mtlD* locus, which clearly indicates the genetic instability of mannitol production in those strains.

By aligning product formation to biomass synthesis, growth-coupled production promises to become a useful strategy to stabilize production from CO_2_ in cyanobacteria [13]. To implement this strategy, product formation needs to be either beneficial to the cells, or become a mandatory process for biomass synthesis. Under such conditions, Darwinian selection will ensure that the producing cells will not be outcompeted by non-producing mutant cells, i.e., the production would be stabilized. Before such rationale can be applied to mannitol production in cyanobacteria, we first want to understand the function that mannitol plays inside the cells that are able to produce this compound. According to literature, mannitol can serve as a compatible solute in selected bacteria, presumably because it will protect cells under high-salt- and/or oxidative stress conditions [14]. Since cyanobacteria natively produce various compatible solutes (e.g., trehalose, glycine betaine, sucrose, glucosyl-glycerol, etc.) to accommodate the consequences of salt stress [15, 16], we want to first experimentally validate whether mannitol can be used to replace the cyanobacterial native compatible solutes under salt stress conditions. If so, mannitol production would be expected to be beneficial for cyanobacterial cells that lack the endogenous compatible solutes in their resistance to salt stress. A mannitol production system would hence be expected to be stable in such cyanobacterial mutants under salt stress conditions.

To implement this idea, the model freshwater cyanobacterium *Synechocystis* sp. PCC6803 (hereafter, *Synechocystis*) was chosen over *Synechococcus* for a few reasons. In addition to being extensively genetically engineered for the production of a variety of biofuels and chemicals [4], *Synechocystis* is the only cyanobacterium in which the molecular mechanism of salt stress has been studied in detail [17]. The genes and biosynthetic pathways of its native compatible solutes (i.e., sucrose and glycosyl-glycerol) under salt stress have been clarified [18], which facilitates their targeted deletion and provides a straightforward approach to introduce a ‘mannitol cassette’ (*mtlD* and *m1p*). Furthermore, the freshwater *Synechocystis* has a much higher sensitivity to salt stress than the marine *Synechococcus* [19, 20]. During high salt acclimation, one would therefore expect more mannitol to be produced in *Synechocystis* than in *Synechococcus*, provided salt tolerance increases with cellular mannitol production. Given these reasons, we have selected *Synechocystis* as the microbial host to be genetically engineered for mannitol production.

In this study, we achieved mannitol production in *Synechocystis* via heterologous gene expression of a cassette composed of the codon-optimized mannitol-1-phosphate-5-dehydrogenase (*mtD*) from *E. coli* and mannitol-1-phosphatase (*m1p*) from *Eimeria tenella*. We have further shown that mannitol can indeed function as a compatible solute, to benefit cell growth especially for the *Synechocystis* mutant strains that have lost the ability to synthesize their endogenous compatible solute(s). Significantly, by adding salt to the growth media, this strategy has been proven capable of stabilizing mannitol production during prolonged cultivations in these latter *Synechocystis* strains.

## Results and discussion

### Engineering mannitol-producing *Synechocystis* strains

**Fig. 1:**
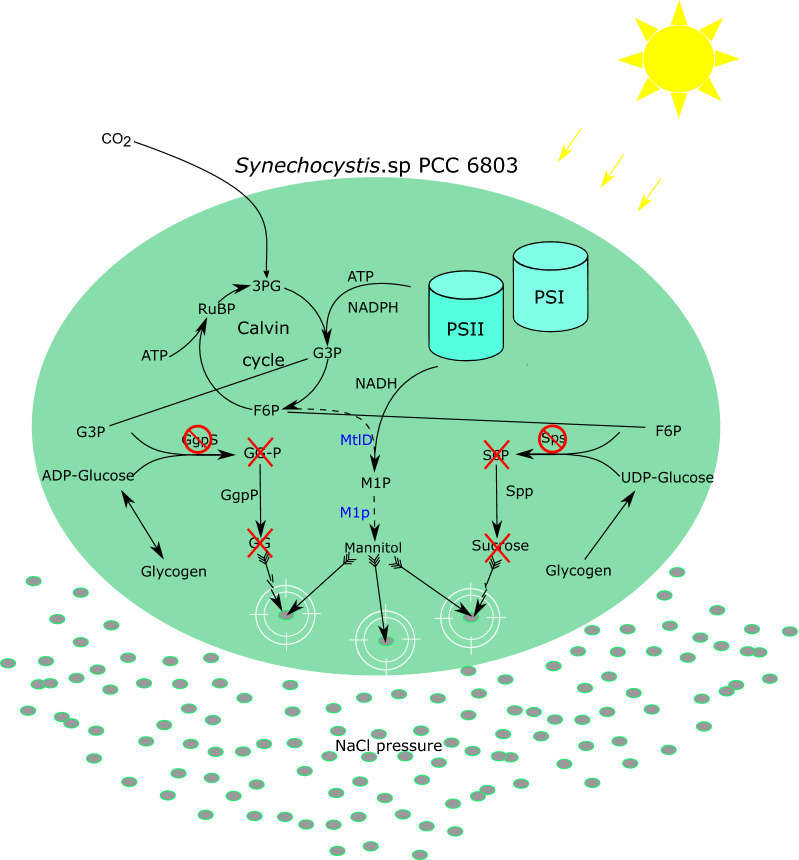
Overview of the engineered biosynthetic pathway to mannitol in the cyanobacterium *Synechocystis* sp. PCC6803. Solid arrows without wings represent native steps in cell; dashed arrows represent introduced pathway of mannitol synthesis; engineered enzymes are shown in blue; red circles with diagonal red line indicate deleted proteins; red cross-lines indicate metabolites that cannot be synthesized. White double circles represent protection against osmotic pressure by compatible solutes or mannitol in the cells. Grey ovals represent salt stress. 3PG, 3-phosphoglycerate; F6P, fructose-6-phosphate; G3P, glyceraldehyde-3-phosphate; M1P, mannitol-1-phosphate; M1p, Mannitol-1-phosphatase (M1Pase; encoded by *m1p* from *E.tenella*); MtlD, Mannitol-1-phosphate dehydrogenase (M1PDH; encoded by *mtlD* from *E. coli*); GgpS, Glucosyl-glycerol phosphate synthase; GG-P, glucosyl-glycerol-phosphate; GgpP, Glucosylglycerol phosphate phosphatase; GG, glucosyl-glycerol; Sps, Sucrose phosphate synthase; S6P, sucrose-6-phosphate; Spp, Sucrose phosphate phosphatase; RuBP, ribulose-1,5-bisphosphate

It has been reported that mannitol can be synthesized from fructose-6 phosphate via the sequential enzymatic reactions catalyzed by mannitol-1-phosphatedehydrogenase (*mtlD*) and mannitol-1-phosphatase (*m1p*) (Fig. 1). All our efforts to construct plasmids containing a functional mannitol cassette under control of the strong constitutive Ptrc1 promoter (a hybrid between the trp and lac UV5 promoters) have failed in *E. coli*, presumably due to the toxicity of certain sugar phosphates [11]. For instance, *E. coli* mutants that lose the function of fructose-1-phosphate dehydrogenase, via mutagenesis, have impaired cell growth due to the intracellular accumulation of fructose-1-phosphate [21]. A similar phenomenon was also observed in *Salmonella typhimurium*, when mannitol was added to the growth medium, cells that lost the function of mannitol dehydrogenase could not grow because of the intracellular accumulation of mannitol-1-phosphate [22]. Hence, instead of using an intact plasmid, a fused and linear DNA fragment, consisting of the homologous regions of *slr0168* (a non-essential hypothetical protein), the mannitol production cassette and a kanamycin resistance cassette, was constructed to be integrated via natural transformation at the neutral site *slr0168* of the *Synechocystis* genome [23]. After several attempts, we obtained a few positive colonies, though a methionine was always stubbornly missing at position 332 of *mtlD* (even though not present in the linear DNA fragment used for the transformation). Nonetheless, since even with this mutated *mtlD*, mannitol production could still be observed, we decided to continue with this construct for the subsequent experiments.

**Fig. 2:**
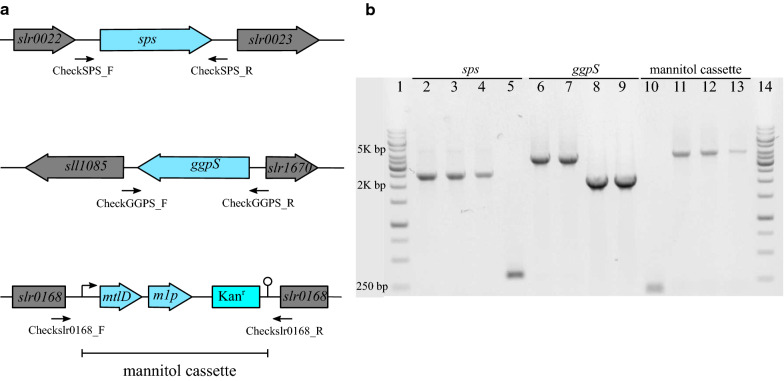
Identification and confirmation genotype by PCR in the *Synechocystis* genome. **a** Map of primer-binding sites. Primers CheckSPS_F and CheckSPS_R were used for segregation analysis of the *sps* locus (PCR products 300 bp in mutant and 2400 bp in WT). Primers CheckGGPS_F and CheckGGPS_R were used to check the *ggpS* locus (PCR products 2100 bp in mutant and 4800 bp in WT). Primers Checkslr0168_F and Checkslr0168_R were used to check the *slr0168* locus (PCR products 5200 bp in mutant and 200 bp in WT). **b** PCR analyses for genotyping of mutants. The chromosomal DNA is from WT (lanes 2, 6 and 10), WT_M (lanes 3,7 and 11), ΔGGPS_M (lanes 4,8 and 12) and ΔCS_M (lanes 5,9 and 13) of *Synechocystis* as a template and primers CheckSPS_F and CheckSPS_R specific for selected genes *sps* (lanes 2 to 5), primers CheckGGPS_F and CheckGGPS_R specific for selected genes *ggpS* (lanes 6 to 9), and primers Checkslr0168_F and Checkslr0168_R specific for selected genes mannitol cassette (lanes 10 to 13) to verify the genotype in the chromosomal DNA of the mutants. Lanes 1 and 14 ladder (1 kb from Fisher Scientific Company). Kan^r^, indicates kanamycin resistance cassette

**Fig. 3:**
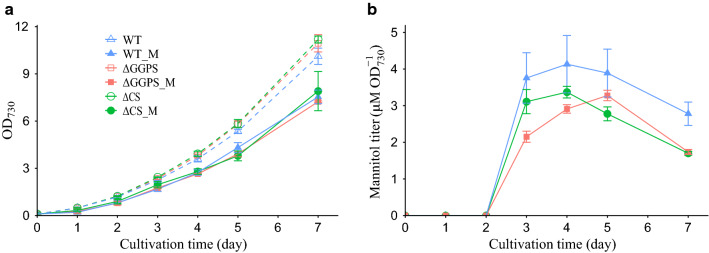
Growth curve and mannitol production of *Synechocystis* mutants in normal BG-11 medium. **a** Growth curve in cultures of WT, WT_M, ΔCS, ΔCS_M, ΔGGPS and ΔGGPS_M **b** Mannitol accumulation in three different mannitol-producing strains WT_M, ΔCS_M and ΔGGPS_M. Symbols: solid triangles, WT_M; solid squares, ΔGGPS_M; solid circles, ΔCS_M; open triangles, WT; open squares, ΔGGPS; open circles, ΔCS. Values represent the average of at least three biological replicates (error bars represent standard deviation). In a, the broken lines connect the data points of the strains lacking the mannitol cassette

This mannitol cassette was expressed in the wild type (WT), ∆*ggpS* (ΔGGPS) and ∆*ggpS*∆*sps* (ΔCS) *Synechocystis* backgrounds, resulting in the strains: WT_M, ΔGGPS_M and ΔCS_M, respectively (Fig. 2a, b and Additional file 1: Table S1). Compared with a previous study in *Synechococcus* [9], this mutated mannitol cassette seems much easier to fully segregate, which occurs for the *slr0168* neutral site of *Synechocystis* in only 5 days under 50 µg mL^−1^ kanamycin. This could be influenced by the lower expression and/or activity level of MtlD due to the mutation on position 332. Next, we monitored the growth of each mutant in regular BG-11 medium. Growth of the strains with a functional mannitol cassette appeared clearly impaired (Fig. 3a). This is due to the burden of mannitol production, for instance because part of the carbon fixed in photosynthesis is not available for biomass formation. Strikingly, impairing the ability to synthesize glycosyl-glycerol, or both sucrose and glycosyl-glycerol, did not lead to any improvement in mannitol production; it even had a negative impact relative to the *Synechocystis* WT background (Fig. 3b and Additional file 1: Table S1).

### Mannitol production confers cells with higher salt tolerance

To test whether mannitol can functionally replace the native compatible solutes of *Synechocystis* to resist high-salt stress, the salt sensitivity of the WT, ΔGGPS and ΔCS mutants, in the absence and presence of the mannitol cassette, was assayed under a wide range of salt stress conditions via a spot assay. Linear gradient NaCl plates were prepared with a salt gradient from 0 to 1 M for the WT and ΔGGPS strains, and from 0 to 0.5 M for the ΔCS strain. The two salt gradients were used because the ΔCS strain is much more sensitive to increasing salt concentrations than the other two strains. This is to such an extent that the salt tolerance conferred by mannitol production can in fact only be clearly noticed for the ΔCS strain when the concentrations are between 0 and 0.5 M. Next, exponentially growing cultures of all strains were diluted to a concentration of 12,500 cells µL^−1^ and 5 µL of each strain was spotted multiple times across the salt gradient on the BG-11 plate. The two different salt gradient plates were incubated at 30 °C under a constant moderate light intensity of ~ 50 µmol photons m^−2^ s^−1^. After about 1 week, the spots became green and the results were then analyzed.

**Fig. 4:**
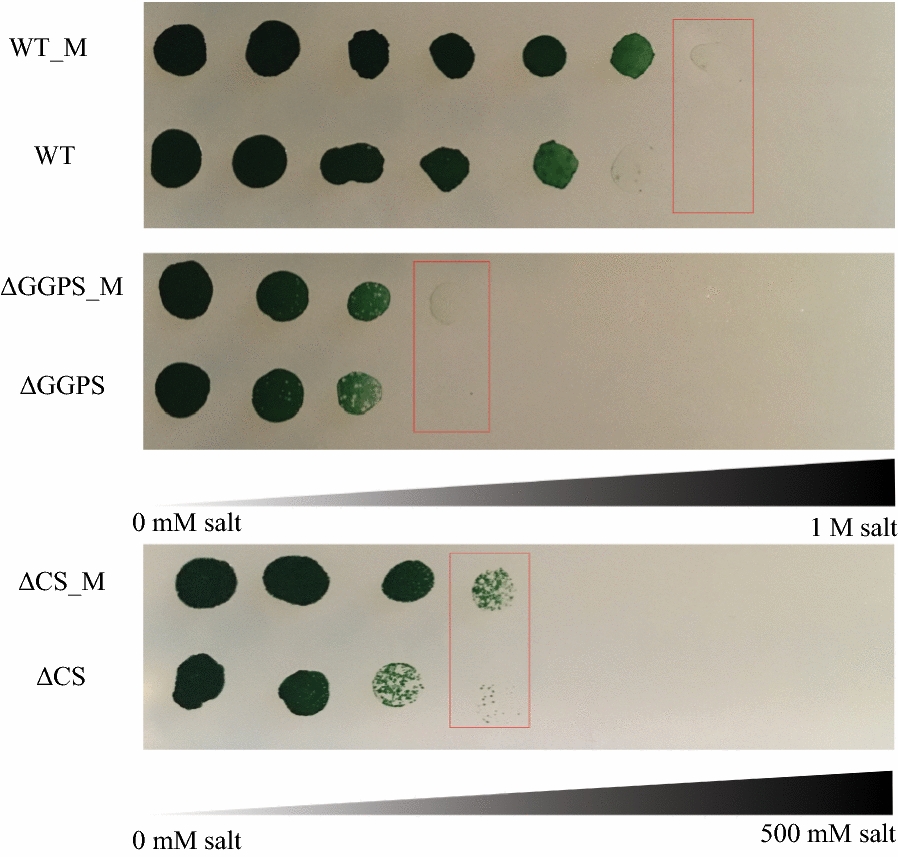
The effect of salt concentrations on the growth of *Synechocystis*. The strains WT, WT_M, ΔGGPS, ΔGGPS_M, ΔCS and ΔCS_M were grown to OD_730_ = 1.0, after which 5 µl of WT, WT_M, ΔGGPS and ΔGGPS_M were transferred to a plate with a linear gradient of NaCl with a maximum salt concentration of 1 M; in addition, 5 µl of ΔCS and ΔCS_M was grown on a plate with a linear gradient with a maximum salt concentration of 0.5 M. The plates were incubated for 12 days at 30 °C. Red rectangles emphasize differences in salt resistance between corresponding strains (i.e., the same genetic background, but with and without the mannitol cassette)

All the strains carrying a mannitol cassette displayed increased salt tolerance (Fig. 4). In the *Synechocystis* WT background, sucrose and glycosyl-glycerol are the two main endogenous compatible solutes that are used by the cells to cope with high salt stress. It is quite interesting to see that even with the native compatible solutes present in the cell, mannitol production still confers cells even higher salt tolerance in the WT background (see top panel of Fig. 4). When the biosynthetic capacity for either glycosyl-glycerol (in the ΔGGPS strain), or both glycosyl-glycerol and sucrose (in the ΔCS strain), was deleted, cells displayed a lower salt tolerance relative to the WT strain. This further supports the functionality of the native compatible solutes to resist salt stress. In the ΔCS_M mutant, mannitol is the only (known) compatible solute remaining. Indeed, mannitol production helped the ΔCS_M mutant to cope with higher salt stress (Fig. 4). Accordingly, we anticipated that mannitol production in *Synechocystis* can be stabilized via salt stress; yet this still needed to be directly tested.

### Salt stress stabilized mannitol production during prolonged cultivations

**Fig. 5:**
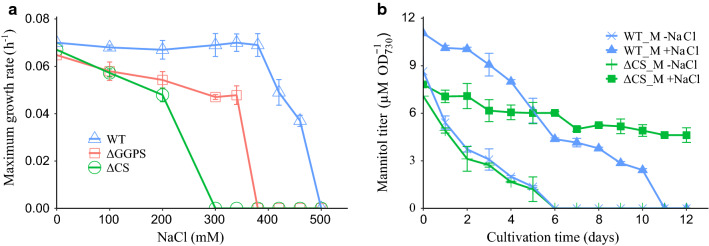
Maximum growth rate for mutants under different salt concentration and mannitol production changed from mannitol producer under specific salt pressures in Multi-cultivator. **a** Growth rate of WT, ΔGGPS and ΔCS as a function of salt concentration. Symbols: open triangles, WT; open squares, ΔGGPS; open circles, ΔCS. Values represent the average of biological duplicates (error bars represent standard deviation). **b** Mannitol production of WT_M and ΔCS_M with and without salt stress in a Multi-cultivator. Symbols: crosses, WT_M grown in the presence of 420 mM NaCl; triangles, WT_M grown without added salt; bars, ΔCS_M grown without added salt; squares, ΔCS_M grown in the presence of 200 mM NaCl. Values represent the average of at least three biological replicates (error bars represent standard deviation)

To test the (genetic) stability of mannitol production, a suitable NaCl concentration has to be selected. This salt concentration should partially inhibit cell growth, but should still allow cells to replicate at a certain rate, such that reverting cells that may arise, but should not be able to take over the population in a relatively short time. Hence, we decided to first determine the growth rate of all the background strains under different NaCl concentrations in a 96-well plate growth assay (Fig. 5a and Additional file 1: Figure S1). As depicted in Fig. 5a, the results obtained showed that the growth rate of the WT strain was hardly affected, at least up to 400 mM NaCl, and then its growth rate gradually decreased until the growth was completely arrested at 500 mM NaCl. For the ΔGGPS strain, which lacks the main native compatible solute glucosyl-glycerol, the growth rate slightly decreased at 350 mM NaCl and suddenly dropped sharply to zero at 400 mM NaCl, and the same trend is observed for the ΔCS. For this latter strain, the growth rate already decreased at 200 mM NaCl and went to zero at 300 mM NaCl.

Based on these results, to check the stability of mannitol production during prolonged cultivation, we decided to select the NaCl concentration that allows 70% of the maximum growth rate. We decided to leave out the ΔGGPS strain, because of possible contribution of sucrose to the salt tolerance of the cells. Hence, 420 mM and 200 mM NaCl were the salt concentrations chosen to test the WT_M and ΔCS_M strains, respectively.

Mannitol production for the WT_M and ΔCS_M strains was monitored with and without salt stress, in prolonged turbidostat cultivations. As shown in Fig. 5b, for both the WT_M and the ΔCS_M strain without salt stress, mannitol production was quickly lost until no mannitol could be measured after only 6 days of cultivation (13.5 generations). This further supports the argument that mannitol production is indeed unstable via common metabolic engineering strategies as previously observed in *Synechococcus* [9]. For the WT_M strain under salt stress, mannitol production gradually dropped to below the detection limit in 11 days (17.1 generations). This phenomenon is to be expected because native compatible solutes are likely to be preferred to resist salt stress, over the exogenous mannitol. In the ΔCS_M strain, mannitol will be the only compatible solute available to the cells to tolerate salt stress. Therefore, mannitol production is expected to be stable in this strain. This is in accordance with what we observed, as throughout the entire experiment (i.e., 12 days, 21.6 generations, see Fig. 5b), only a slight drop in mannitol production was observed in the ΔCS_M strain, especially during days 6 and 7. We speculate that this slight decrease might be due to the fact that cells within the population are selected when mannitol production is fine-tuned to the amounts required by the environment imposed. These results indicate that by exploiting salt stress as a selection pressure, one can stabilize mannitol production from CO_2_ in *Synechocystis*.

### Characterization of mutations that are selected by the phenotypic instability

**Fig. 6:**
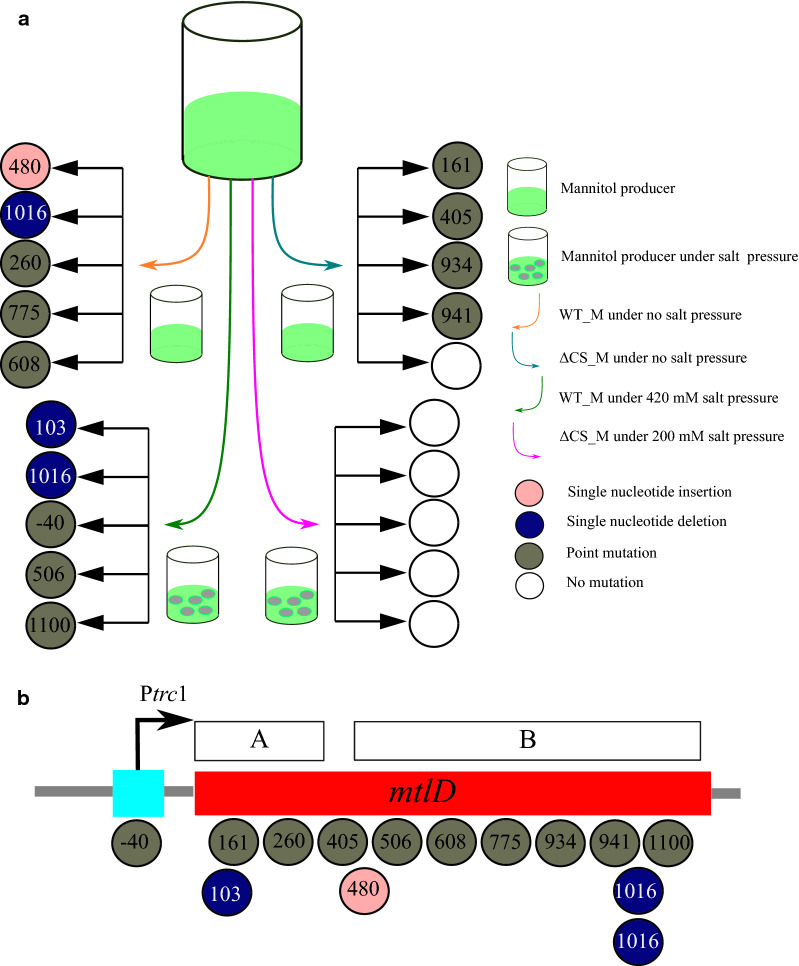
Characterization of mutations underpinning phenotypic instability surrounding mannitol production under non-stabilizing conditions in Multi-cultivator. **a** Schematic representation on the analysis of genetic stability of mannitol producers in the Multi-cultivator. The colored arrows in the center refer to the four combinations of a relevant strain (i.e., WT and ΔCS_M) plus cultivation condition (i.e., in the presence and absence of NaCl). Mannitol production capacity of WT_M and ΔCS_M under no salt condition was completely eliminated within only 6 days. WT_M produces decreasing amounts of mannitol, until it ceases to do so, during the first 10 days while under 420 mM salt. The mannitol production from ΔCS_M under 200 mM salt was found to be the most stable, which could still be detected after 12 days. The grey ovals in green color indicate salt pressure. For further details, see Results and Discussion. **b** Schematic overview of all mutations observed in the *mtlD*-part of the mannitol cassette of the mannitol-producing strains. The numbers in the circles refer to the position of a mutation in the promoter region or in the reading frame of MtlD. “A” in the bar above the reading frame of MtlD represents the Rossmann domain, and “B” the c-terminal domain. In MtlD, the two domains are linked via a linker region

Mannitol production from WT_M and ΔCS_M under no salt condition in the multi-cultivator was completely absent after only 13.5 generations, which corresponds to only 6 days of cultivation. Under 420 mM salt, the WT_M produced decreasing amounts of mannitol until it completely ceased to do so after 17.1 generations in 11 days of cultivation. In sharp contrast, the ΔCS_M under 200 mM salt stress displayed remarkably stable mannitol production, which could still be observed after 12 days cultivation in the multi-cultivator, corresponding to over 21.6 generations. To clarify the molecular mechanism(s) behind the phenotypic instability of mannitol production, we decided to sequence the mannitol cassette of cultures derived from prolonged turbidostat cultivation experiments. From each strain and growth condition, 5 single colonies (i.e., 20 single colonies in total) were isolated when the mannitol productions from their responding culture were completely disrupted and their mannitol-producing cassette was amplified by PCR and sent for sequencing (Additional file 1: Table S2). Indeed, the results obtained showed that for those cultivations in which mannitol production was lost, various mutations could be detected, either leading to a truncated, non-functional, MtlD protein, or to impairment of its enzyme activity. All mutations found could be grouped in one of three types: single nucleotide insertions (SNI), single nucleotide deletions (SND) and single point mutations (PM) (see Additional file 1: Table S2 and Fig. 6). Among 14 mutations found in the strains that lost the ability to synthesize mannitol, 13 mutations occurred in *mtlD* reading frame and one was in its (Ptrc1) promoter region. All these mutants of different mannitol producers cultured under various salt conditions were re-cultivated in the shake flasks, and none of them showed the ability of producing mannitol (data not shown). These results indicated that mannitol production was disrupted due to malfunction of MtlD and that mannitol-1-phosphate accumulation might be harmful to cells, even though the underlying mechanism is still unclear [11]. We know the MtlD protein contains two pfam domains, the mannitol dehydrogenase C-terminal domain (pfam08125) and mannitol dehydrogenase Rossmann domain (pfam01232). The Rossmann domain is specific for binding NAD(P)^+^ and contains the conserved consensus motif G-x-G-x-x-G. The C-terminal domain contains conserved residues which bind the substrate fructose-6-phosphate, thus facilitating the catalytic reaction of the enzyme [24]. Our data revealed that three of the mutations were distributed over the Rossmann domain, 9 were in the C-terminal domain, including two identical mutations isolated independently, and one was in the linker region. It is important to note that one of the isolates from the ΔCS_M population, cultured under no salt condition, showed no mutation while its culture had stopped producing mannitol within 6 days. This result suggests that though a very small proportion of this population might still have the ability to synthesize mannitol, its ratio of non-mutated over mutated cells must be too low to make detectable amounts of mannitol by enzymatic assay.

In sharp contrast, no mutation in the mannitol cassette was found in the ΔCS_M strain during the entire prolonged cultivation under salt stress. This observation corroborates the result that cells also maintained a continuous mannitol producing phenotype under such conditions (Fig. 6a) and leads us to conclude that mannitol production in *Synechocystis* can be stabilized by salt stress, in mutants lacking the pathways for synthesis of the two endogenous osmoprotectants. In addition, by purposely weakening the native abilities of cells to tolerate salt stress [25, 26], this strategy could allow more mannitol to be synthesized to compensate the loss of salt tolerance ability. Such could allow that mannitol production could be even further sustainably enhanced.

## Conclusions

Mannitol production was achieved in a freshwater cyanobacterium, a derivative of *Synechocystis*, this time conferring the producing strain with increased salt tolerance. With this approach we have tackled the major hurdle to mannitol production in cyanobacteria—genetic instability—by specifically aligning the production of a target compound with a fitness gain for the producing cells. Given the osmoprotectant properties of mannitol, we targeted the native osmotic stress response of *Synechocystis*, by replacing the synthesis of the endogenous compatible solutes by mannitol production. This strategy has been shown to be very successful, resulting in a production system for mannitol directly from CO_2_ with increased stability. The principle developed here, i.e., of coupling product formation to increased fitness under mild stress conditions, can potentially be applied to other host/product combinations as well. In future work it is important to take this principle of product-mediated stress protection already into account during the metabolic engineering design stage, as this engineering is likely to involve the identification and deletion of native stress response genes.

## Methods

### Strains and culture conditions

Strains of *E. coli* were grown on Lysogeny Broth (LB) liquid medium at 37 °C in a shaking incubator at 200 rpm, or on the solid LB plates with 1.5% agar. Antibiotics were added to LB liquid medium or to solid plates, with the appropriate concentration as follows: kanamycin (50 µg mL^−1^) or ampicillin (100 µg mL^−1^), either separately or in combination.

*Synechocystis*, a glucose-tolerant wild type, obtained from D. Bhaya, University of Stanford, Stanford CA, was cultivated in a modified BG-11 medium enriched with 25 mM PIPPS buffer (pH 8.0) [27] at 30 °C, either in a shaking incubator at 120 rpm, or on solid BG-11 plates, supplemented with 1.5% agar and 0.3% (w/v) sodium thiosulphate. The cells were grown with white light of moderate intensity (~ 50 µmol photons m^−2^ s^−1^), except when indicated. To construct *Synechocystis* mutants, kanamycin or nickel was added separately into BG-11 liquid medium or solid plates, with the appropriate concentration as follows: kanamycin (50 µg mL^−1^) and/or nickel (20 µM). The culture density was monitored by determining the optical density at a wavelength of 730 nm (OD_730_).

### Gene synthesis with codon optimization

The gene sequence of mannitol-1-phosphate-5-dehydrogenase (*mtlD*) from *E. coli* (NCBI Reference Sequence: NC_000913.3) and mannitol-1-phosphatase (*m1p*) from *Eimeria tenella* (NCBI Reference Sequence: AF032462.1) was taken directly from the NCBI database. Codon optimization was performed based on the codon usage table compiled for *Synechocystis* (https://www.kazusa.or.jp/codon/). The genes were synthesized by GenScript Biotech Corp, and were ligated to the pHKH and pUC57 plasmids, respectively [28] resulting in pHKHmtlD and pUCm1p.

### Plasmids and *Synechocystis* mutant construction

To obtain marker-less deletion strains, the genes encoding sucrose phosphate synthase (*sps*) and glucosyl-glycerol phosphate synthase (*ggpS*) in the genome of *Synechocystis* were deleted with a counter-selection approach [29]. For each gene deletion, two plasmids are needed: the first one contains only the upstream and the downstream sequences of the region to be deleted, hereafter referred as the homologous regions, while the second plasmid contains an extra selection cassette flanked by both homologous regions. The selection cassette consists of a gene conferring kanamycin resistance to the host, as well as a toxic gene (*mazF*), under transcriptional control of the tightly regulated nickel-inducible promoter PnrsB. To construct each of the two plasmids, ~ 1 kb of each upstream and downstream homologous region of either *sps* or *ggpS* was individually PCR-amplified from the genome of *Synechocystis*, using Herculase II Fusion DNA Polymerase (Agilent Technologies). After gel purification, each set of fragments of an upstream and downstream homologous region was fused by overlap PCR, and the entire fused fragment was then further amplified by PCR. After the fused fragment was gel purified, an extra adenosine was added to the 3′ ends of these fragments and it was then ligated to the pFL-AN-T vector [30], resulting in plasmids pFL-AN2 (Δ*sps*) and pFL-AN4 (Δ*ggpS*), respectively. Because an XbaI restriction site was introduced via the primers during overlap PCR, the selection cassette, if provided with an XbaI site on both ends, can be easily inserted into pFL-AN2 (Δ*sps*) and pFL-AN4 (Δ*ggpS*), resulting in pFL-AN1 (Δ*sps*) and pFL-AN3 (Δ*ggpS*), respectively.

The *mtlD* and *m1p* genes were PCR amplified from pHKHmtlD and pUCm1p, respectively. Initially, we attempted to clone these genes using *E. coli* as a shuttle host. To correctly express *mtlD* in *Synechocystis*, the weaker promoter PnrsB was used to control the expression level of *mtlD* in *Synechocystis*, but repeatedly failed. This result surprised us, as PnrsB is from the nickel response system (*nrs* [31]), and is regarded as one of the weakest promoters in this host in the absence of an inducer. This result indicated the difficulty of high expression levels of MtlD, and that this might become a bottleneck for the synthesis of large amounts of mannitol in *Synechocystis*. So we decided to bypass the usage of a shuttle host. Instead we chose to fuse these two fragments together with the kanamycin resistance gene, and the upstream and downstream homologous regions of *slr0168*, via overlap PCR. The resulting mannitol cassette plus resistance marker was placed under control of the Ptrc1 promoter. The fused fragment was sequenced to check for the absence of mutations and then used directly for transformation of the chromosome of *Synechocystis* at the neutral site present in locus *slr0168.*

It takes two rounds of natural transformation of *Synechocystis* to achieve a markerless gene deletion of either *sps* or *ggpS*. The first round is to fully integrate the selection cassette into the chromosome through homologous recombination, while the second round is to completely remove the selection cassette. The method used for natural transformation was essentially as described previously [32]. In brief, 1 ml *Synechocystis* cultures grown in a shake flask to an OD_730_ of ~ 0.4 were harvested and concentrated by centrifugation at 5000 rpm for 5 min to a volume of 200 µL. Then, plasmid was added to the concentrated cells at 10 µg mL^−1^, followed by 5 h’ incubation in moderate white light, in a shaking incubator at 150 rpm. After incubation, cells were spread onto a commercial membrane (Pall Corporation, USA) resting on a BG-11 plate without antibiotic pressure. After a 24-h incubation in the 30 °C incubator under constant white light illumination, the membrane was transferred onto a new BG-11 plate with 50 µg mL^−1^ kanamycin. Single colonies appeared after approximately 12 days. The segregation status of mutants was confirmed by PCR, using the appropriate primers. When a mutant was confirmed to be fully segregated, a second round of transformation with a plasmid containing only the upstream and downstream homologous region was performed. The selection was then based on the resistance to nickel as only the colonies with the selection cassette fully removed can survive on the plates with nickel. The protocol for transformation of the DNA fragment containing the mannitol cassette and the kanamycin resistance fragment integrated at the *slr0168* site was similar to the protocol mentioned above. Full segregation of this construct was achieved by propagations in the presence of kanamycin. All the mutants were confirmed by PCR and the primers that were used are listed in Additional file 1: Table S3. The confirmed mutants were routinely stored at −80 °C in BG-11 medium supplemented with 20% (v/v) glycerol.

### Growth rate determination under salt stress

To measure the growth rate for each strain under salt stress, we monitored the growth of each strain in a 96-well plate under a range of NaCl concentrations, from 0 to 500 mM. A preculture was prepared by inoculating cells from glycerol stocks directly into shake flasks containing liquid BG-11 medium and cultivated in the incubator with shaking of 120 rpm under continuously white light of moderate intensity (~ 50 µmol photons m^−2^ s^−1^). Once the precultures reached OD_730_ = 1, a total volume of 1 mL of a pre-culture was harvested and inoculated in each well of a 48-well plate, supplemented with 50 mM NaHCO_3_ plus increasing salt concentrations, ranging from 0 to 500 mM. After 2 days, the final optical density of the cultures in the 48-well plate was measured using a SPECTROstar Nano Microplate Photometer (BMG LABTECH GmbH, Germany) at 730 nm. To initiate experiments with a 96-well plate, pre-cultures acclimated to the corresponding salt stress in the 48-well plate were used for inoculation. This is to prevent the prolonging of the lag-phase of growth, resulting from the addition of salt, such that the same stage of cell growth under each condition can be extracted from the data for growth rate calculation. Pre-cultures were then diluted using 50 mM NaHCO_3_ and the respective concentration of NaCl in BG-11 to an initial OD of 0.05. Plates were incubated under constant white light illumination, with shaking at 600 rpm. Growth was monitored every 2 h within the 36-h incubation in the plate reader, to reliably calculate the maximum growth rate under each condition, the first 6 consecutive data points (from time 0 to 10 h) were used by fitting a linear function through the natural logarithm of the OD. The slope of the linear function was computed and designated as the growth rate. A representative set of growth curves in the 96 well plate together with the data points for growth rate calculation for all the strains is presented in Additional file 1: Fig. S1.

### Plate assay with a linear NaCl gradient

The method for making linear salt gradient plates has been described previously by [33]. In brief, BG-11 media containing agar, with and without NaCl (either 0.5 M or 1 M), were individually prepared. When making linear salt gradient plates, one side of a square petri dish was lifted and the plate was filled with BG-11 agar without NaCl. After the agar solidified, the plate was placed in a horizontal position and BG-11 agar with NaCl was poured on top of the first layer. To test the salt tolerance of all the mutants, the cells in *Synechocystis* cultures were first counted using a Casy 1 Model TTC cell counter (Schärfe System GmbH, Reutlingen, Germany) with a 60-µm diameter capillary, and diluted to a total cell number of 12,500 cells µL^−1^. A 5 µL culture from each mutant, grown with 200 mM salt, was spotted on the linear gradient plates containing a 0 to 0.5 M or a 0 to 1 M NaCl concentration gradient. Visible, green colonies appeared within 1 week.

### Measurements of extracellular mannitol concentrations

Extracellular mannitol concentrations were determined in the supernatant collected from *Synechocystis* cultures using a D-Mannitol-L-Arabitol Assay Kit (Megazyme) [9]. Cells from shake-flask cultures were removed by centrifugation at 12,000 rpm for 1 min. Then, 100 µL of the supernatant samples was used for mannitol measurement according to manufacturer’s instructions. In this assay, the conversion of the mannitol present in the sample to mannose—catalyzed by mannitol dehydrogenase—is stoichiometrically coupled to the conversion of NAD^+^ to NADH. This leads to an increase of absorbance at 340 nm that can be measured using a plate reader (BMG FLUOstar OPTIMA Microplate Reader). For mannitol quantification, the assay was calibrated with a standard curve (from 3 to 100 µM mannitol) obtained under the same conditions.

### Turbidostat cultivation

The phenotypic stability of mannitol-producing strains was studied with the turbidostat mode of a Multi-Cultivator (MC1000-OD, PSI, Czech Republic). In this cultivation mode, *Synechocystis* populations can be kept at a fixed biomass density by continuously diluting cultures with fresh BG-11 medium without antibiotic, while simultaneously taking out an identical volume of cultured cells. Accordingly, cells in a turbidostat are under continuous selection for maximal specific growth rate. For this we used a modified Multi-Cultivator with additional pumps (Reglo ICC, ISMATEC, Germany) and controlled by the “pycultivator” software package [13]. Pre-cultured cells were transferred into 8 independent cylindrical vessels of a multi-cultivator, filled with BG-11 medium with 0, 200 mM or 420 mM NaCl to an initial OD_730_ of 0.05. OD_730_ was measured every 5 min. Once the threshold of OD_730_ was reached (OD_730_ = 1) cell cultures were automatically diluted by 5% (v/v) with fresh BG-11 medium and the same volume of culture as the volume of medium just added, was discarded; all under control of “pycultivator”. All the cultures in the Multi-Cultivator were exposed to continuous white light with an intensity of 100 µmol photons m^−2^ s^−1^ OD^−1^. The genetic stability of each strain was assessed by mannitol production. Growth rate was calculated by fitting a linear function through the natural logarithm of the OD_730_ during each “growth-dilution” cycle. Samples for extracellular mannitol quantification were periodically taken during the cultivation period. The variation of growth rate and mannitol productivity throughout the whole experiment was then calculated relative to the initial values obtained at the beginning of each specific experiment.

### Sequencing of the mannitol cassette

To check the sequence, the mannitol cassette for the occurrence of (a) spontaneous mutation(s), single colonies from each independent Multi-Cultivator culture were first isolated. This is achieved by taking 5 µL of culture and re-streaking the cells on BG-11 agar plates. After picking a single colony and inoculating it into liquid BG-11 medium, genomic DNA was extracted as previously described [34] and used as a template to amplify the mannitol cassette by PCR, using the high-fidelity Herculase II Fusion DNA Polymerase. The PCR product was then purified using MSB Spin PCRapace (STRATEC Molecular, Germany) and sent for sequencing (Macrogen) using the primers listed in Additional file 1: Table S3.

## Supplementary information

**Additional file 1.** Additional tables and figure.

## Data Availability

The datasets generated during this study are included in this published article and its additional file.
