# Supplementary material for: Using osmotic stress to stabilize mannitol production in Synechocystis sp. PCC6803
Source: Biotechnol Biofuels. 2020 Jul 2;13:117. doi: 10.1186/s13068-020-01755-3 (PMC7331161; doi:10.1186/s13068-020-01755-3)
Supplement: Supplementary file 1 — Additional file 1. Additional tables and figure. [file 13068_2020_1755_MOESM1_ESM.pdf]

Additional file 1

Table S1. Comparison of mannitol production in cyanobacteria

Species of cyanobacteria	Extracellular mannitol concentration (mM)	Cultivation time (days)	Final OD_{730}	Synthesis pathway	Reference
Synechococcus sp. PCC7002	4.5	12	13	MtlD and M1p	(1)
Synechococcus sp. PCC7002	~0.55	52	~22	M1PDH/M1Pase*	(2)
Synechocystis sp. PCC6803	0.021	7	7.5	MtlD and M1p	This study

*, Fusion protein

Table S2. Plasmids and strains used in this study

Plasmid and strains	Description	Reference
pFL-AN	BioBrick "T" vector with AvrII and NheI on each side	(3)
pFL-AN1	pFL-AN derivate, $Amp^r Km^r$, containing *sll0045(sps)* gene upstream homologous region, selection cassette (*mazF*) and downstream homologous region	In this work
pFL-AN2	pFL-AN derivate, Amp^r, containing *sll0045(sps)* gene upstream and downstream homologous regions	In this work
pFL-AN3	pFL-AN derivate, $Amp^r Km^r$, containing *sll1566 (ggpS)* gene upstream homologous region, selection cassette (*mazF*) and downstream homologous region	In this work
pFL-AN4	pFL-AN derivate, Amp^r, containing *sll1566* (*ggpS*) gene upstream and downstream homologous regions	In this work
pHKH015	Integration vector on *slr0168* containing *ldh* (from *B. subtilis*) and *sth* (from *P. aeruginosa*)	In this work
pHKHmtlD	plasmid containing *mtlD*	In this work
pUC57m1p	plasmid containing *m1p*	In this work
WT	*Synechocystis* sp. PCC6803 wild type	(4)
ΔGGPS	*Synechocystis* sp. PCC6803 *ggpS* gene knock out	In this work
SPS	*Synechocystis* sp. PCC6803 *sps* gene knock out	In this work
ΔCS	*Synechocystis* sp. PCC6803 *ggpS* and *sps* double gene knock out mutant	In this work
WT_M	Mannitol cassette under Ptrc1 promoter on the WT background	In this work
ΔGGPS_M	Mannitol cassette under Ptrc1 promoter on the ΔGGPS background	In this work
SPS_M	Mannitol cassette under Ptrc1 promoter on the SPS background	In this work
ΔCS_M	Mannitol cassette under Ptrc1 promoter on the ΔCS background	In this work

Table S3. Summary of all the mutations in the mannitol cassette, identified after prolonged cultivation in the Multi-Cultivator.

Mutation type	Position (start from ATG)	Translation analysis	Enzyme	Cultivation condition
Single nucleotide insertion (SNI)	480	Translation 161 a.a (220 extra codons after stop)	Mannitol dehydrogenase (C-terminal domain)	WT_M under no salt
Point mutation (PM)	260	a.a 87 (A to V)	Mannitol dehydrogenase (Rossmann domain)	WT_M under no salt
Point mutation (PM)	775	a.a 259 (M to L)	Mannitol dehydrogenase (C-terminal domain)	WT_M under no salt
Point mutation (PM)	608	a.a 203 (A to D)	Mannitol dehydrogenase (C-terminal domain)	WT_M under no salt
Single nucleotide deletion (SND)	1016	Translation 339 a.a (42 extra codons after stop)	Mannitol dehydrogenase (C-terminal domain)	WT_M under no salt
Point mutation (PM)	-40		Promoter	WT_M under 420mM salt
Single nucleotide deletion (SND)	1016	Translation 339 a.a (42 extra codons after stop)	Mannitol dehydrogenase (C-terminal domain)	WT_M under 420mM salt
Point mutation (PM)	1100	a.a 367 (T to S)	Mannitol dehydrogenase (C-terminal domain)	WT_M under 420mM salt
Single nucleotide deletion (SND)	103	Translation 34 a.a (347 extra codons after stop)	Mannitol dehydrogenase (Rossmann domain)	WT_M under 420mM salt
Point mutation (PM)	506	a.a 169 (I to N)	Mannitol dehydrogenase (C-terminal domain)	WT_M under 420mM salt
Point mutation (PM)	161	a.a 54 (N to T)	Mannitol dehydrogenase (Rossmann domain)	SG_M under no salt
Point mutation (PM)	405	a.a 135 (I to M)	Mannitol dehydrogenase (Linker region)	SG_M under no salt
Point mutation (PM)	941	a.a 314 (S to T)	Mannitol dehydrogenase (C-terminal domain)	SG_M under no salt
Point mutation (PM)	934	a.a 312 (G to L)	Mannitol dehydrogenase (C-terminal domain)	SG_M under no salt

Table S4 Primers used in this study

Primer name	Sequence	Purpose
Hom1SPS_F	5'-ACATCCCCTCGCTTAACTCC-3'	Amplification of homologous region upstream the *sps* gene
XbaIHom1SPS_R	5'-GTAATTTGTAAAACTTtctagaCCAGCCGAAATCATCGAGAAC-3'	Amplification of homologous region upstream the *sps* gene and addition of an XbaI restriction site at the 3´
XbaIHom2SPS_F	5'-GATGATTTCGGCTGGtctagaAAGTTTACAAATTACTAT-3'	Amplification of homologous region downstream the *sps* gene and addition of an XbaI restriction site at the 5´
Hom2SPS_R	5'-TGGACCTATATCGCCGCTTT-3'	Amplification of homologous region downstream the *sps* gene
Hom1GGPS_F	5'-TCCTTTCCCAACGAAACAAG-3'	Amplification of homologous region upstream the *ggps* gene
XbaIHom1GGPS_R	5'-CTGCAGTTTCTAGACCATATGAAAATCAGCGGTCTCCAAAATC-3'	Amplification of homologous region upstream the *ggps* gene and addition of an XbaI restriction site at the 3´
XbaIHom2GGPS_F	5'-CATGGTCTAGAAACTGCAGGCGATCGCCAATGCCAGTTG-3'	Amplification of homologous region downstream the *ggps* gene and addition of an XbaI restriction site at the 5´
Hom2GGPS_R	5'-TATCCACAAACGCTTCCACA-3'	Amplification of homologous region downstream the *ggps* gene
CheckSPS_F	5'-TTGAAGGAGTTTATGGCCCC-3'	Check deletion of *sps* gene
CheckSPS_R	5'-TAACTCAGAGATTGCGGCCA-3'	Check deletion of *sps* gene
CheckGGPS_F	5'-AACGTACTAAAATGCCCCGG-3'	Check deletion of *ggps* gene
CheckGGPS_R	5'-GGCGACAGGGTTTGAAACAA-3'	Check deletion of *ggps* gene
Ptrc1Hom1slr0168_F	5'-TCTCCACGCTGAATTAGAACA-3'	Amplification of homologous region upstream the *slr0168* gene and promoter *Ptrc*1
Ptrc1Hom1slr0168_R	5'- ATGTCATTTCTCCTCTTTAATG -3'	Amplification of homologous region upstream the *slr0168* gene and promoter *Ptrc*1
MtlD_F	5'-CATTAAAGAGGAGAAATGACATATGAAAGCTTTGCACTTTGG -3'	Amplification of optimized *mtlD* and fused with promoter *Ptrc*1
MtlD_R	5'-ATGTCATTTCTCCTCTTTAATGCTAGCTTATTATTGCATGGCCTTATAGGCCGT -3'	Amplification of optimized *mtlD* and fused with optimized *m1p*
M1p_F	5'-ACGGCCTATAAGGCCATGCAATAATAAGCTAGCATTAAAGAGGAGAAATGACAT-3'	Amplification of optimized *m1p* and fused with optimized *mtlD*
M1p_R	5'-CGGTTTCGCGTTGGGAATCA-3'	Amplification of optimized *m1p*
Kan_F	5'-TGATTCCCAACGCGAAACCGTAATAACCTAGGTCACACTGGCT-3'	Amplification of kanamycin resistance gene and fused with optimized *m1p*
Kan_R	5'- CGCTGAGGTCTGCCTCGTGAAG-3'	Amplification of kanamycin resistance gene
Hom2slr0168_F	5'-TTCACGAGGCAGACCTCAGCGGTCGACCTCGAGAGACCAAGCCC-3'	Amplification of homologous region downstream the *slr0168* gene and fused with kanamycin resistance gene
Hom2slr0168_R	5'- AACCCAGATGGCATCAGC-3'	Amplification of homologous region downstream the *slr0168* gene
Checkslr0168_F	5'-TGTCGCCGCTAAGTTAGA-3'	Check insertion/segregation at the *slr0168* site
Checkslr0168_R	5'-CTGTGGGTAGTAAACTGGC-3'	Check insertion/segregation at the *slr0168* site

Figure S1

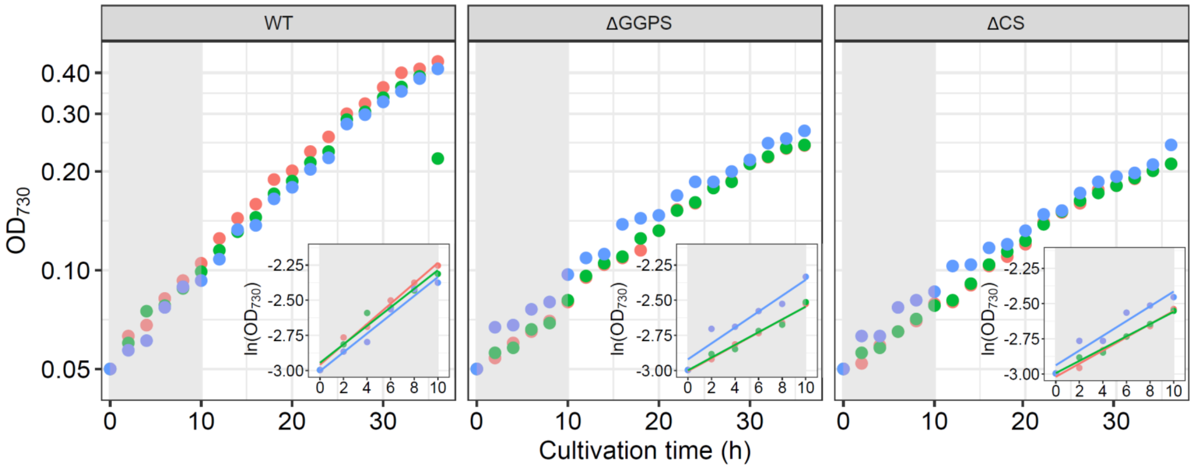

A representative set of growth curves of the strain WT, ΔGGPS and ΔCS in growth medium with 200 mM salt added, in a 96 well plate. Each color represents one replicate. The data with the grey background were extracted for growth rate calculation by fitting a linear function through the natural logarithm of the OD_{730} (indicated as the inset of each plot). The slope of the linear function was computed and designated as the growth rate.

Reference:

1. Jacobsen JH, Frigaard N-U. Engineering of photosynthetic mannitol biosynthesis from CO2 in a cyanobacterium. Metab Eng. 2014 Jan 1;21:60–70.

2. Madsen MA, Semerdzhiev S, Amtmann A, Tonon T. Engineering Mannitol Biosynthesis in *Escherichia coli* and *Synechococcus* sp. PCC 7002 Using a Green Algal Fusion Protein. ACS Synth Biol. 2018;7(12):2833–40.

3. Du W, Jongbloets JA, Guillaume M, van de Putte B, Battaglino B, Hellingwerf KJ, et al. Exploiting Day- and Night-Time Metabolism of *Synechocystis* sp. PCC 6803 for Fitness-Coupled Fumarate Production around the Clock. ACS Synth Biol. 2019 Oct 18;8(10):2263–9.

4. Ng W-O, Grossman AR, Bhaya D. Multiple Light Inputs Control Phototaxis in *Synechocystis* sp. Strain PCC6803. J Bacteriol. 2003 Mar 1;185(5):1599 LP – 1607.
